# Mitochondrial Fusion by M1 Promotes Embryoid Body Cardiac Differentiation of Human Pluripotent Stem Cells

**DOI:** 10.1155/2019/6380135

**Published:** 2019-09-19

**Authors:** Jarmon G. Lees, Anne M. Kong, Yi C. Chen, Priyadharshini Sivakumaran, Damián Hernández, Alice Pébay, Alexandra J. Harvey, David K. Gardner, Shiang Y. Lim

**Affiliations:** ^1^St. Vincent's Institute of Medical Research, VIC, Australia; ^2^Monash University, VIC, Australia; ^3^Centre for Eye Research Australia, Royal Victorian Eye and Ear Hospital, VIC, Australia; ^4^Department of Medicine and Surgery, University of Melbourne, VIC, Australia; ^5^Department of Anatomy and Neuroscience, University of Melbourne, VIC, Australia; ^6^School of BioSciences, University of Melbourne, VIC, Australia

## Abstract

Human induced pluripotent stem cells (iPSCs) can be differentiated *in vitro* into *bona fide* cardiomyocytes for disease modelling and personalized medicine. Mitochondrial morphology and metabolism change dramatically as iPSCs differentiate into mesodermal cardiac lineages. Inhibiting mitochondrial fission has been shown to promote cardiac differentiation of iPSCs. However, the effect of hydrazone M1, a small molecule that promotes mitochondrial fusion, on cardiac mesodermal commitment of human iPSCs is unknown. Here, we demonstrate that treatment with M1 promoted mitochondrial fusion in human iPSCs. Treatment of iPSCs with M1 during embryoid body formation significantly increased the percentage of beating embryoid bodies and expression of cardiac-specific genes. The pro-fusion and pro-cardiogenic effects of M1 were not associated with changes in expression of the *α* and *β* subunits of adenosine triphosphate (ATP) synthase. Our findings demonstrate for the first time that hydrazone M1 is capable of promoting cardiac differentiation of human iPSCs, highlighting the important role of mitochondrial dynamics in cardiac mesoderm lineage specification and cardiac development. M1 and other mitochondrial fusion promoters emerge as promising molecular targets to generate lineages of the heart from human iPSCs for patient-specific regenerative medicine.

## 1. Introduction

Induced pluripotent stem cells (iPSCs) are patient-specific somatic cells that have been reprogrammed to a pluripotent state carrying the same genetic makeup as the parental cells and show great promise for advancing autologous cell therapies. Cardiomyocytes derived from iPSCs are a renewable source of cells for cell-based therapies to treat heart disease, as well as for drug screening and disease modelling. Therapeutic success in the field of human heart disease will rely on our understanding of the molecular and cellular events that govern cardiac differentiation of iPSCs. While the transcriptional drivers of mesodermal cardiac differentiation of iPSCs have been described [1], the role of mitochondrial bioenergetics and morphology that underpin cardiomyocyte lineage specification is only just beginning to be uncovered [2–5].

PSCs exhibit a low oxidative and high glycolytic nature relative to most differentiated cell types [6–10], reflected in their mitochondria which are small and punctate and display a perinuclear localization [3, 11]. It has recently emerged that as PSCs exit pluripotency and begin to differentiate into ectoderm, mesoderm, and endoderm, they undergo a brief metabolic surge [12, 13] followed by germ layer-specific metabolic patterning [8, 13]. Specifically, mesodermal cardiac differentiation initiates an increase in mitochondrial mass and dispersal of elongated and networked mitochondria throughout the cytoplasm [7, 14].

Mitochondria continuously undergo fission and fusion processes under the regulation of a group of evolutionarily conserved mitochondrial fission and fusion proteins, respectively [15]. Mice deficient in mitochondrial fission (Drp1 and Mff) or fusion (Mfn1, Mfn2, and Opa1) proteins develop cardiac defects highlighting the importance of mitochondrial dynamics in cardiac lineage specification and cardiovascular homeostasis [16–18]. Likewise, mouse embryonic stem cells deficient in mitochondrial fusion proteins Mfn2 and Opa1 show impaired cardiac differentiation [19]. We have recently shown that cardiac differentiation of human iPSCs can be induced by knockdown or inhibition of the mitochondrial fission protein DRP1 (*DNM1L*) [7]. These studies suggest that stimulating mitochondrial fusion dynamics may allow for more efficient and robust mesodermal cardiac differentiation of iPSCs.

M1 is a hydrazone compound shown to promote mitochondrial fusion in mouse embryonic fibroblasts (MEFs) [20]. The pro-fusion effect of M1 is dependent on basal fusion activity as M1 does not promote mitochondrial fusion in Mfn1/2- or Opa1-double-knockout (KO) MEFs [20]. The pro-mitochondrial fusion effect of M1 has also been shown in human macrophages, rat pancreatic cells, and rat hippocampal neurons, where M1 rescues mitochondrial dysfunction and fragmentation induced by either cholesterol [21, 22] or amyloid beta exposure [23].

In light of these findings, we aimed to investigate the potential cardiogenic effect of M1 in human iPSCs, hypothesizing that stimulating mitochondrial fusion with M1 would promote mesodermal cardiac differentiation of iPSCs. To test this hypothesis, we treated human iPSCs with M1 under both pluripotency-maintaining and cardiac differentiation culture conditions and in both 2D and 3D cultures. For mechanistic insights, we examined the expression of mitochondrial ATP synthase subunits in response to M1 treatment as well as the kinase interaction profile of M1.

## 2. Results

### 2.1. M1 Promotes Fusion of Mitochondria in Human iPSCs

**Figure 1:**
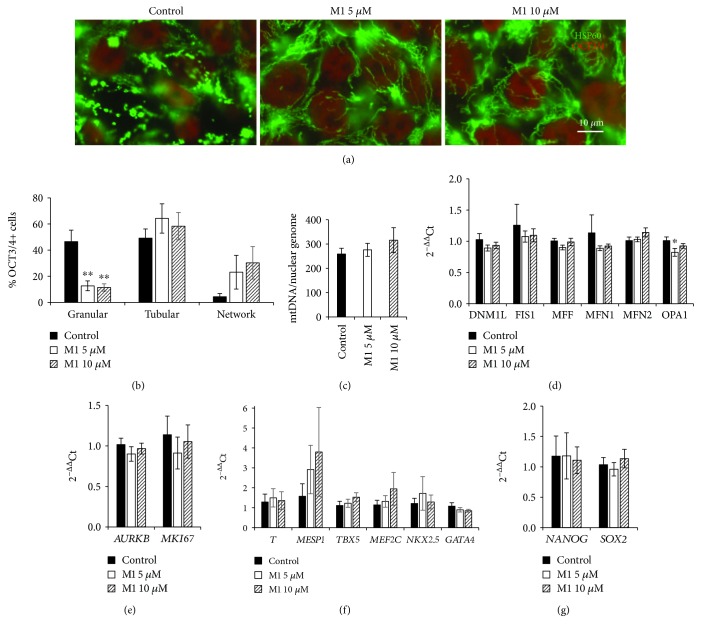
M1 stimulates mitochondrial fusion in human iPSCs (iPSC-Foreskin-2 cell line) without inducing mesendodermal differentiation. (a) Mitochondrial morphology of human iPSCs, indicated by HSP60 staining. (b) Percentage of OCT3/4+ cells with different mitochondrial morphologies (*n* = 4). (c) Number of mitochondrial genomes per nuclear genome (*n* = 6). (d–g) mRNA expression of mitochondrial fission and fusion markers (d), cell proliferation markers (e), mesodermal cardiac transcription factors (f) ,and pluripotency markers (g) in human iPSCs treated with either DMSO vehicle control (control) or M1 at 5 or 10 *μ*M for 48 hours (*n* = 7). Data are expressed as mean ± SEM. ^∗^*P* < 0.05, ^∗∗^*P* < 0.01 vs. control by one-way paired ANOVA with Dunnett's post hoc test.

To investigate whether M1 influences mitochondrial morphology in human iPSCs, undifferentiated iPSCs were cultured in TeSR-E8 pluripotency-maintaining medium with M1 for 48 hours. M1 at 5 *μ*M has been shown to elicit a morphological and functional mitochondrial response in rat neurons and mouse embryonic fibroblasts (MEFs) [20, 23]. Treatment with both 5 and 10 *μ*M of M1 significantly reduced the proportion of granular mitochondria in OCT3/4^+^ cells in the iPSC-Foreskin-2 cell line (Figures 1(a) and 1(b)). However, the proportions of pluripotent iPSCs with tubular or networked mitochondria were not significantly increased. Loss of punctate mitochondrial morphology was not accompanied by a change in the mitochondrial DNA (mtDNA) copy number, which remained stable with M1 treatment in the iPSC-Foreskin-2 cell line (Figure 1(c)). mRNA levels of mitochondrial fission genes were not altered; however, there was a small but significant reduction in the expression of the mitochondrial fusion gene *OPA1* in cells treated with 5 *μ*M of M1 in the iPSC-Foreskin-2 cell line (Figure 1(d)). Treatment with M1 did not significantly affect the expression of cell proliferation markers (*AURKB* and *MKI67*, Figure 1(e)), mesodermal cardiac markers (*T*, *MESP1*, *TBX5*, *MEF2C*, *NKX2.5*, and *GATA4*, Figure 1(f)), endoderm markers (*CDH1* and *AFP*, Supplementary Fig. [Supplementary-material supplementary-material-1]), ectoderm marker (*TUBB3*, Supplementary Fig. [Supplementary-material supplementary-material-1]), or pluripotency markers (*NANOG* and *SOX2*, Figure 1(g)) in iPSCs cultured in TeSR-E8 pluripotency-maintaining medium. The ectoderm marker *PAX6* was decreased following M1 treatment at 5 *μ*M but not 10 *μ*M (Supplementary Fig. [Supplementary-material supplementary-material-1]).

To demonstrate the reproducible effect of M1 on human iPSCs, we cultured another human iPSC line, CERA007c6, in pluripotency-maintaining medium with 1, 5, 10, and 50 *μ*M of M1 for 48 hours. Consistent with the results observed in the iPSC-Foreskin-2 cell line, treatment with 1, 5, 10, or 50 *μ*M of M1 did not significantly affect the expression of fission or fusion genes (*DNM1L*, *FIS1*, *MFF*, *MFN1*, *MFN2*, and *OPA1*, Supplementary Fig. [Supplementary-material supplementary-material-1]), mesodermal cardiac markers (*TBX5* and *GATA4*, Supplementary Fig. [Supplementary-material supplementary-material-1]), endoderm markers (*CDH1* and *AFP*, Supplementary Fig. [Supplementary-material supplementary-material-1]), or ectoderm markers (*PAX6* and *TUBB3*, Supplementary Fig. [Supplementary-material supplementary-material-1]) in CERA007c6 iPSCs.

**Figure 2:**
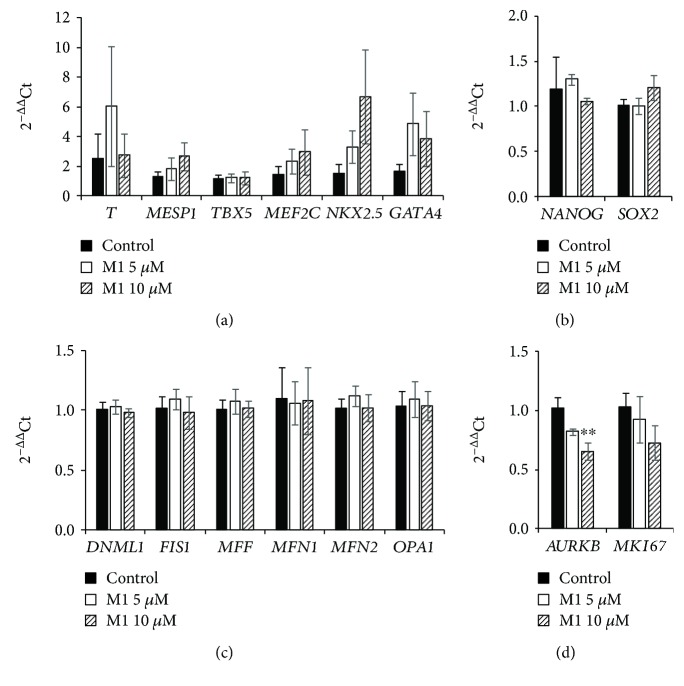
Gene expression of human iPSCs (iPSC-Foreskin-2 cell line) treated with M1 in differentiation medium. (a–d) mRNA of mesodermal cardiac transcription factors (a), pluripotency markers (b), mitochondrial fission and fusion markers (c), and cell proliferation markers (d) in human PSCs treated with either DMSO vehicle control (control) or M1 at 5 or 10 *μ*M in RPMI+B-27 medium for 48 hours. *n* = 5. Data are expressed as mean ± SEM. ^∗∗^*P* < 0.01 vs. control by one-way paired ANOVA with Dunnett's post hoc test.

To determine if the TeSR-E8 pluripotency-maintaining medium was masking the effect of M1, iPSC-Foreskin-2 iPSCs were cultured for 48 hours in mesodermal differentiation medium that consisted of RPMI basal medium and B-27 supplement (RPMI+B-27 medium), with or without 5 or 10 *μ*M of M1 or a vehicle control (0.05% of DMSO). Compared with iPSCs cultured in TeSR-E8, culture in RPMI+B-27 medium without M1 for 48 hours significantly decreased the expression of the pluripotency marker *SOX2* (Supplementary Fig. [Supplementary-material supplementary-material-1]) and increased the expression of the mesoderm transcription factor *MESP1* (Supplementary Fig. [Supplementary-material supplementary-material-1]) indicating a departure from pluripotency. Mitochondrial fission genes *DNM1L* and *MFF* and fusion gene *OPA1* were significantly downregulated, while the fission gene *FIS1* was increased (Supplementary Fig. [Supplementary-material supplementary-material-1]). The cell proliferation marker *MKI67* and cytokinesis marker *AURKB* were not significantly affected (Supplementary Fig. [Supplementary-material supplementary-material-1]). In iPSCs cultured in RPMI+B-27 medium, treatment with M1 did not significantly affect the mRNA levels of the assessed mesodermal or cardiac transcription factors (Figure 2(a)), nor were pluripotency markers *NANOG* and *SOX2* different from the DMSO control (Figure 2(b)). Mitochondrial fission and fusion genes were similarly unchanged (Figure 2(c)). The expression of the cell proliferation marker *AURKB*, but not *MKI67*, was significantly downregulated in cells treated with 10 *μ*M of M1 (Figure 2(d)). These data indicate that while M1 promotes mitochondrial fusion in human iPSCs, it does not induce cardiac mesoderm differentiation in iPSCs cultured in a 2D format.

### 2.2. Promoting Mitochondrial Fusion with M1 Enhances Embryoid Body-Based Cardiac Differentiation

**Figure 3:**
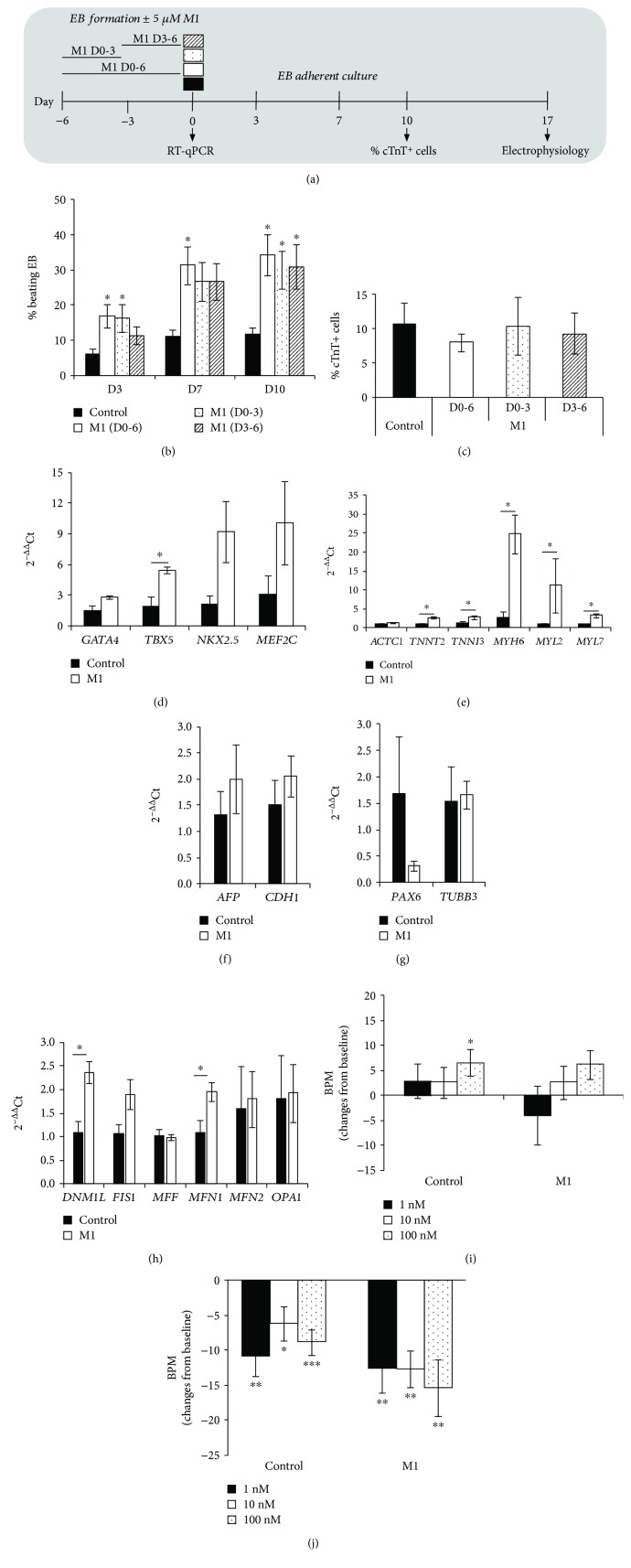
M1 promotes cardiac differentiation of human iPSCs (iPSC-Foreskin-2 cell line). (a) Schematic of embryoid body- (EB-) based cardiac differentiation protocol with 5 *μ*M of M1 treatment regimen. (b) Effect of M1 on the percentage of beating EBs (*n* = 8). (c) Percentage of cardiac troponin T-positive (cTnT^+^) cells in individual beating EBs at day 10 postplating (*n* = 8). (d–h) mRNA expression of mesodermal cardiac transcription factors (d), cardiac-specific muscle proteins (e), endoderm lineage markers (f), ectoderm lineage markers (g), and mitochondrial fission and fusion markers (h) in human iPSCs treated with DMSO (control) or 5 *μ*M M1 for 6 days during EB formation (*n* = 4). (i, j) Changes in the beating rate of cardiomyocytes derived from control or M1 groups following treatment with isoproterenol hydrochloride (isoprenaline: 1–100 nM (i)) or carbamylcholine (carbachol: 1–100 nM (j)) (*n* = 10). Data are expressed as mean ± SEM. ^∗^*P* < 0.05, ^∗∗^*P* < 0.01, and ^∗∗∗^*P* < 0.001 vs. control by one-way ANOVA with Dunnett's post hoc test (b, c, i, j) and by paired Student's *t*-test (d–h).

We have recently shown that inhibiting mitochondrial fission with Mdivi-1 enhances cardiac differentiation of iPSCs in a 3D embryoid body (EB) model [7]. To evaluate the impact of promoting mitochondrial fusion during 3D cardiac differentiation, human iPSC-Foreskin-2 iPSCs undergoing a 6-day EB-based spontaneous differentiation protocol were treated with M1 (Figure 3(a)). Treatment of iPSCs with 5 *μ*M M1 throughout EB formation resulted in a significant 2- to 3-fold increase in the percentage of beating EBs at days 3, 7, and 10 postplating (Figure 3(b)). Treatment duration slightly impacted the procardiogenic effects of M1 as the treatment during the first 3 days of EB formation, but not during the second 3 days, significantly increased the beating EB rate at 3 days postplating. However, equivalent increases in the percentage of beating EBs were achieved by all M1 treatment groups at 10 days postplating (Figure 3(b)). Despite the increase in the percentage of beating EBs, the percentage of cardiac troponin T-positive cardiomyocytes within each beating EB at day 10 postplating was similar among all treatment groups (Figure 3(c)). Directed cardiac differentiation from iPSCs using 5 *μ*M of M1 throughout the 6 days of EB formation was confirmed by quantitative PCR analysis, whereby the cardiac transcription factor *TBX5* (Figure 3(d)) as well as cardiac structural and contractile proteins *TNNT2*, *TNNI3*, *MYH6*, *MYL2*, and *MYL7* (Figure 3(e)) were significantly upregulated in 6-day-old EBs. Endoderm (Figure 3(f)) and ectoderm (Figure 3(g)) lineage markers were unchanged due to M1 treatment. mRNA transcripts for the mitochondrial fission protein *DNM1L* and mitochondrial fusion protein *MFN1* were significantly upregulated in M1-treated EBs (Figure 3(h)).

The electrophysiological properties of EBs were evaluated at 17 days postplating using microelectrode arrays (MEA). Extracellular field potentials recorded a contraction rate of 70 ± 3 bpm in the control group and 76 ± 6 bpm in the M1-treated group. Isoproterenol (Figure 3(i)) and carbamylcholine (Figure 3(j)) treatment resulted in a concentration-dependent positive and negative chronotropic response, respectively, in both the control and M1-treated beating EBs. Field potential duration was similar between groups at all concentrations (data not shown). These findings indicate that while M1 was able to promote EB-based cardiac differentiation, M1 did not influence the electrophysiological properties of cardiomyocytes generated from human iPSCs.

### 2.3. M1 Does Not Increase ATP Synthase Subunit Expression in Human iPSCs

**Figure 4:**
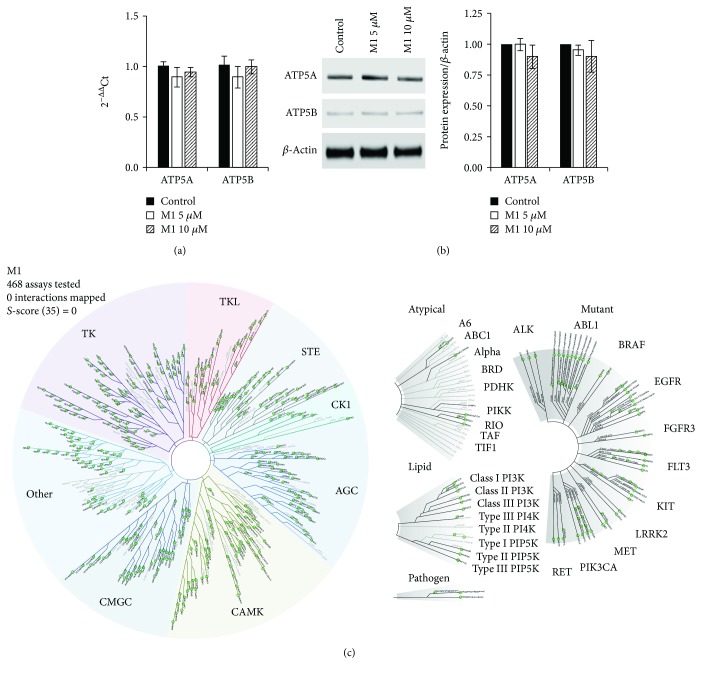
M1 does not alter ATP synthase subunit expression or interact with any of the 468 human protein kinases screened. (a) mRNA (*n* = 7) and (b) protein (*n* = 6) expression of ATP synthase subunits in human iPSCs (iPSC-Foreskin-2 cell line) cultured in TeSR-E8 and treated with M1 for 48 hours. (c) Kinase interaction map of M1 at 10 *μ*M with 468 human protein kinases. Zero interactions with a binding score of ≥35% relative to DMSO were identified. Data are expressed as mean ± SEM and analyzed by one-way paired ANOVA with Dunnett's post hoc test.

It has been shown that M1 rescues the expression of Atp5a/b that is lost in Mfn1/2-KO MEFs, suggesting that the pro-fusion effects of M1 may be associated with increased expression of the catalytic ATP synthase subunits *α* and *β* [20]. To evaluate whether M1 might act mechanistically through ATP synthase in iPSCs, we examined the expression of ATP5A and ATP5B mRNA transcripts (Figure 4(a)) and protein (Figure 4(b)) in response to 5 and 10 *μ*M of M1 in iPSC-Foreskin-2 iPSCs cultured in pluripotency-maintaining medium for 48 hours. Despite the clear change in mitochondrial morphology observed under the same treatment conditions (Figure 1(a)), ATP synthase subunits *α* and *β* were not changed at either the mRNA or protein level. We confirmed this using CERA007c6 iPSCs cultured in pluripotency-maintaining medium with 1, 5, 10, or 50 *μ*M of M1 for 48 hours and observed no effect at any concentration on *ATP5A* or *ATP5B* mRNA levels (Supplementary Fig. [Supplementary-material supplementary-material-1]). Relative to cells cultured in TeSR-E8 medium, iPSC-Foreskin-2 iPSCs cultured in RPMI+B-27 medium showed upregulation of *ATP5B* mRNA (Supplementary Fig. [Supplementary-material supplementary-material-1]). However, treatment with M1 significantly reduced the expression of *ATP5B* at 10 *μ*M and had no effect on the expression of *ATP5A* in iPSC-Foreskin-2 iPSCs cultured in RPMI+B-27 medium (Supplementary Fig. [Supplementary-material supplementary-material-1]), nor did it affect *ATP5A* or *ATP5B* expression in 3D embryoid body-based cardiac differentiation (Supplementary Fig. [Supplementary-material supplementary-material-1]).

We further explored the potential involvement of protein kinases that M1 might interact with using a high-throughput ATP-independent kinase assay. Kinase screening against a panel of 468 human kinases covering over 80% of the human kinome did not reveal any thermodynamic interaction with M1 (Figure 4(c) and Supplementary [Supplementary-material supplementary-material-1]). Overall, these data demonstrate that promotion of mitochondrial fusion by M1 does not require increased expression of ATP5A or ATP5B, nor does M1 interact with any of the 468 protein kinases tested to promote mitochondrial fusion.

## 3. Discussion

Mitochondria are highly plastic in their morphology. They constantly change their shape by fusion and fission processes that are important for the maintenance of cellular functions including metabolism, proliferation, apoptosis, signalling, and determining cell fate [3]. As PSCs differentiate, their mitochondria shift from a perinuclear, fragmented morphology to a dispersed, fused network, a process that is reversed when somatic cells are reprogrammed to iPSCs [24, 25]. This phenomenon highlights the importance of mitochondrial morphology in regulating stem cell fate [2–5, 26]. In this study, we uncovered the pro-cardiogenic effects of hydrazone M1, a small molecule that promotes mitochondrial fusion. M1 fuses the native fragmented mitochondria in human iPSCs and promotes their differentiation into an early mesodermal cardiac lineage.

Mitochondria of human PSCs closely resemble those of the embryonic inner cell mass [3]. They are spherical in shape with clear matrices and few peripheral arched cristae, a morphology that is proposed to support the pluripotent state [3]. On the other hand, mitochondria of somatic cells are typically filamentous with many transverse cristae to support a higher level of oxidative metabolism [6, 27]. Currently, M1 is the only known small molecule capable of promoting mitochondrial fusion in fragmented mitochondria. Our findings demonstrate for the first time that M1 is capable of fusing the fragmented mitochondria present in human iPSCs. The shift from punctate, perinuclear mitochondria to more dispersed, filamentous mitochondria within 48 hours did not coincide with a change in pluripotency or differentiation, specifically in mesodermal cardiac markers. This is consistent with the timing of metabolic and lineage marker acquisition during differentiation, as changes in mitochondrial morphology, glycolysis, and mitochondrial metabolism precede the loss of pluripotency markers and the upregulation of lineage markers [24, 28]. Similarly, during reprogramming, glycolysis is acquired prior to pluripotency [25, 29]. This might suggest that, rather than being a consequence, a metabolic state drives reprogramming and differentiation of PSCs.

As PSCs differentiate and commit to either ectoderm, mesoderm, or endoderm, a lineage-specific metabolism is acquired [30]. Ectoderm differentiation entails the suppression of mitochondrial metabolism and a concomitant increase in glycolytic metabolism [13]. In contrast, mesoderm and endoderm lineages require increased mitochondrial oxidative metabolism and active suppression of glycolytic metabolism [8]. Consequently, mesodermal cardiac differentiation from PSCs necessitates a shift from a glycolytic metabolism in PSCs to an oxidative metabolism in cardiomyocytes, which includes increases in mitochondrial mass, mtDNA copies, and elongated mitochondria [7, 14, 31]. Supporting this shift in metabolism is a coordinated decrease in mitochondrial fission genes *Dnm1l* and *Mtp18* and an increase in the fusion gene *Mfn2* as murine embryonic stem cells differentiate into cardiomyocytes [32]. The requirement for mitochondrial fusion proteins in cardiac differentiation has been demonstrated previously in mouse embryonic stem cells where knockdown of *Mfn2* and *Opa1* impaired their differentiation into beating cardiomyocytes [19]. We have previously shown reduced expression of DRP1 (*DNM1L*) in differentiated cardiomyocytes compared to undifferentiated human iPSCs, and either genetic knockdown of DRP1 or pharmacological inhibition of DRP1 with Mdivi-1 promotes cardiomyocyte differentiation of iPSCs [7]. To complement these findings, the present study demonstrates that promoting mitochondrial fusion with M1 similarly increases the efficiency of early cardiac differentiation from human iPSCs. This may suggest a role for promoting mitochondrial fusion in priming human iPSCs for mesoderm and endoderm differentiation, given the requirement for increased oxidative phosphorylation by these lineages [8].

M1 promoted cardiac differentiation of human iPSCs in 3D but not 2D cultures. While the 2D culture allows for greater control over directed cardiac differentiation, a 3D differentiation method using foetal bovine serum-containing media allows spontaneous cardiac differentiation of pluripotent stem cells by mimicking the physiological embryonic cardiac development processes, including the dynamic cardiomyocyte and noncardiomyocyte interactions [33, 34]. Therefore, M1 is unlikely to have the ability to directly activate the cardiac transcriptional programmes needed in the 2D directed cardiac differentiation method, but the pro-mitochondrial fusion effect of M1 might be sufficient to facilitate the spontaneous cardiac differentiation process of iPSCs cultured in 3D embryoid bodies.

In the present study, M1 decreased the mitochondrial fusion protein *OPA1* in the 2D culture of the human iPSC-Foreskin-2 cell line despite a shift towards fusion in iPSC mitochondria (Figure 1(d)). The reason for this is unclear although the M1-induced OPA1 reduction was not observed in the CERA007c6 iPSC line (Supplementary Fig. [Supplementary-material supplementary-material-1]). A previous study has highlighted an important role of mitochondrial fusion in mediating the cardiogenesis of pluripotent stem cells that reduction of *OPA1* and *MFN2* levels impairs cardiac differentiation of murine embryonic stem cells [19]. Conversely, cardiac differentiation of embryonic stem cells is associated with reduced expression of *OPA1* [35] and M1 increased *DNM1L* and *MFN1*, but did not alter the *OPA1* gene expression in the 3D culture of human iPSCs in the present study (Figure 3(h)). These findings might suggest that the changes in *OPA1* in the 2D culture of the human iPSC-Foreskin-2 cell line treated with M1 are unlikely to be related to the pro-cardiogenic effect of M1 observed in the 3D culture of iPSCs.

The mechanism by which M1 promotes the fusion of mitochondria remains unclear but has been suggested to involve the catalytic *α* and *β* subunits of ATP synthase [20, 36]. KO of Mfn1 and Mfn2 in MEFs causes a loss of Atp5a/b expression and fragmentation of the mitochondria, while treatment with M1 rescues both Atp5a/b expression and mitochondrial morphology [20]. Overexpression of either Atp5a or Atp5b in Mfn1- and Mfn2-KO MEFs similarly restores the filamentous mitochondrial morphology. Significantly, the pro-mitochondrial fusion effect of M1 has been shown to require basal fusion activity, as no mitochondrial elongation was observed in Mfn1/2-double-KO MEFs treated with M1 [20]. Moreover, M1 only promotes fusion in fragmented mitochondria and does not promote mitochondrial hyperfusion in wild-type MEFs [20]. The present study showed that M1 supplementation caused a loss of the punctate, fragmented mitochondrial morphology in human iPSCs, in the absence of a change in ATP5A or ATP5B mRNA or protein. These data suggest that the catalytic core of ATP synthase is unlikely to be the mechanism by which M1 promotes mitochondrial fusion.

We observed an increase in *ATP5B* mRNA levels within 48 hours of iPSC differentiation (Supplementary Fig. [Supplementary-material supplementary-material-1]), consistent with the well-established shift to elongated mitochondria and upregulation of mitochondrial OXPHOS that takes place during mesoderm differentiation [7, 8, 14]. This may suggest that ATP synthase, specifically the *β* subunit, could be involved in the acquisition of filamentous mitochondria and oxidative metabolism that occurs as iPSCs remodel their metabolism throughout the process of mesoderm differentiation. In support of this role for the ATP synthase *β* subunit, a recent report has shown that activation of ATP5B can promote mitochondrial fission and fusion dynamics in human embryonic kidney 297T cells [37].

The mechanisms underlying the pro-mitochondrial fusion and pro-cardiogenic effect of M1 remain elusive. In an attempt to identify potential mechanistic targets of M1, a high-throughput screening of a library of human protein kinases was performed. Although M1 did not significantly interact with any of the 468 human protein kinases in our kinase profiling assay, this finding does not preclude the possibility that M1 may interact with other protein classes such as GTPases, ion channels, nuclear receptors, and transcription factors. For example, the sarco/endoplasmic reticulum calcium-ATPase [38], nuclear coactivator PGC-1*β* [39], calcineurin [19], nuclear factor erythroid 2-related factor-2 (NRF2) [40], microRNA-106a [41], and microRNA-376b-3p [42] have been shown to interact with mitochondrial fission and fusion proteins to regulate cell fate and function.

In conclusion, stimulating mitochondrial fusion with the small molecule M1 promotes embryoid body-based cardiac differentiation of human iPSCs, highlighting the important role of mitochondrial dynamics in mesoderm lineage specification and efficient cardiac development. It is likely that not only mitochondrial morphology but also mitochondrial metabolism will need to be optimized to achieve the most efficient differentiation of human iPSCs.

## 4. Materials and Methods

### 4.1. Human iPSC Culture and Cardiac Differentiation

The human iPS-Foreskin-2 cell line, kindly provided by James A. Thomson (University of Winconsin) [43], was propagated on a feeder layer of mitotically inactivated human foreskin fibroblasts (HFF:D551; ATCC, VA, USA) in Dulbecco's modified Eagle's medium (DMEM)/F-12 GlutaMAX medium supplemented with 20% knockout serum replacement, 0.1 mM 2-mercaptoethanol, 0.1 mM nonessential amino acids, 50 U/mL penicillin/streptomycin (all from Thermo Fisher Scientific, VIC, Australia), and 20 ng/mL recombinant human fibroblast growth factor-2 (Merck Millipore, CA, USA). Spontaneous *in vitro* differentiation of iPSCs was induced through the formation of embryoid bodies (EBs) in suspension as previously described [44]. Briefly, EBs were formed by mechanically dissecting undifferentiated iPSC colonies maintained on a feeder layer into approximately 0.2 mm^2^ pieces using the sharp edge of a flame-pulled capillary. Pieces were transferred onto low attachment plates and cultured in suspension for 6 days in differentiation medium containing DMEM/F-12 GlutaMAX medium supplemented with 20% foetal bovine serum (Sigma-Aldrich, MO, USA), 0.1 mM 2-mercaptoethanol, 0.1 mM nonessential amino acids, and 50 U/mL penicillin/streptomycin. During EB formation, cells were treated with either 0.05% DMSO or 5 *μ*M M1 from days 0-6, days 0-3, or days 3-6. On day 6 (day 0 postplating), EBs were transferred to 48-well tissue culture plates precoated with 0.1% gelatin (Sigma-Aldrich) and cultured in differentiation medium. The percentage of contractile EBs was measured as the number of EBs that showed spontaneous contraction divided by the total number of EBs plated.

For the 2D monolayer cell culture, human iPSCs (iPSC-Foreskin-2 and CERA007c6 [45] cell lines) were maintained on vitronectin-coated plates in TeSR-E8 medium (Stem Cell Technologies, VA, Canada) according to the manufacturer's protocol. For RNA and imaging, 50,000 cells/cm^2^ were seeded onto Matrigel- (Corning, MA, USA) coated plates or coverslips in TeSR-E8 medium supplemented with 10 *μ*M Y-27632 (Tocris Bioscience, Bristol, UK). After 1 day when the cells were ~60% confluent, the cells were treated with either 0.05% DMSO (as a vehicle control) or M1 at 1, 5, 10, or 50 *μ*M in TeSR-E8 for 48 hours before harvesting. For non-pluripotency-maintaining conditions, iPSCs were seeded as described above in TeSR-E8 for 1 day and then cultured in RPMI medium supplemented with 1x B-27 supplement (Thermo Fisher Scientific) containing either 0.05% DMSO or M1 at 5 or 10 *μ*M for 48 hours.

### 4.2. Microelectrode Array Recordings

Extracellular field potential recording of the beating colonies was performed using the microelectrode array (MEA) recording system (Multichannel Systems, Reutlingen, Germany). Beating EBs at day 10 postplating were transferred onto MEA plates precoated with 0.1% gelatin and 10 *μ*g/mL fibronectin. Responsiveness to isoproterenol hydrochloride (1–100 nM, Sigma-Aldrich) and carbamylcholine (1–100 nM, Sigma-Aldrich) was determined 4–6 days later at 37°C in Krebs-Ringer buffer (composition in mM: 125 NaCl, 5 KCl, 1 Na2HPO4, 1 MgSO4, 20 HEPES, 5.5 glucose, and 2 CaCl2; pH 7.4). Each cell cluster was treated with all drugs in random order, and cells were allowed to recover to their baseline contraction in fresh Krebs-Ringer buffer in between drug treatments. Extracellular field potentials were recorded at baseline and 2 minutes after the addition of drugs. Data were analyzed offline with MC Rack version 4.3.5 software for the beating rate, RR interval, and extracellular field potential duration (FPD) as previously described [7, 44, 46]. FPD measurements were normalized (corrected FPD, cFPD) with the Bazzet correction formula: cFPD = FPD/√(RR interval).

### 4.3. Immunocytochemistry

Immunocytochemistry was performed on cells using the following primary antibodies: Oct3/4 (20 *μ*g/mL, mouse monoclonal IgG; Santa Cruz Biotechnology, TX, USA), cardiac troponin T (cTnT, 2 *μ*g/mL, mouse monoclonal IgG; Abcam, MA, USA), and Hsp60 (1.58 *μ*g/mL, rabbit polyclonal IgG, Abcam). Following overnight incubation with primary antibodies at 4°C, cells were then immunostained with a species-specific secondary antibody: Alexa Fluor 488 goat anti-mouse IgG, Alexa Fluor 488 goat anti-rabbit IgG, or Alexa Fluor 594 goat anti-mouse IgG (10 *μ*g/mL; Thermo Fisher Scientific). Cells were counterstained with DAPI (1 *μ*g/mL; Thermo Fisher Scientific) for nuclear staining and mounted with a fluorescence mounting agent (Dako, Victoria, Australia). Images were acquired with a BX-61 Olympus fluorescence microscope (Tokyo, Japan). For quantitative assessment of mitochondrial morphology, Oct3/4-positive cells were categorically characterized as having either granular (punctate), tubular (elongated), or networked (reticulated) mitochondria, and at least 500 cells were counted per independent biological experiment. For quantitative assessment of cardiomyocyte differentiation, spontaneously beating colonies at 10 days postplating were trypsinized into single-cell suspension with 0.25% trypsin-EDTA, spun onto coated glass slides (4 minutes at 900 rpm; Shandon Cytospin 4, Thermo Fisher Scientific), and immunostained with a cardiac troponin T antibody followed by Alexa Fluor 488 goat anti-mouse IgG. Cells were counterstained with DAPI and mounted with a fluorescence mounting agent. Images were taken with a BX-61 Olympus fluorescence microscope, and at least 500 cells were counted.

### 4.4. Real-Time Quantitative PCR (RT-qPCR)

RNA was extracted from cells using TRI Reagent (Thermo Fisher Scientific) followed by RNA precipitation with chloroform and isopropanol (Sigma-Aldrich). cDNA was synthesized using the high-capacity cDNA reverse transcription kit on 1 *μ*g of RNA (Applied Biosystems, CA, USA). qPCR was carried out using TaqMan Universal master mix, the 7900HT Fast Real-Time PCR system, and TaqMan gene expression assays (Applied Biosystems) for *GAPDH* (Hs03929097_g1), *T* (Hs00610080_m1), *MESP1* (Hs00251489_m1), *TBX5* (Hs00361155_m1), *GATA4* (Hs00171403_m1), *NKX2.5* (Hs00231763_m1), *MEF2C* (Hs00231149_m1), *ACTC1* (Hs01109515_m1), *TNNT2* (Hs01109515_m1), *TNNI3* (Hs00165957_m1), *MYH6* (Hs01101425_m1), *MYL2* (Hs00166405_m1), *MYL7* (Hs01085598_g1), *AFP* (Hs01040598_m1), *CDH1* (Hs01023894_m1), *PAX6* (Hs01088112_m1), *TUBB3* (Hs00801390_s1), *AURKB* (Hs00945855_g1), *MKI67* (Hs01032443_m1), *NANOG* (Hs04260366_g1), *SOX2* (Hs01053049_s1), *DNM1L* (Hs00247147_m1), *FIS1* (Hs00211420_m1), *MFF* (Hs00697394_g1), *MFN1* (Hs00966851_m1), *MFN2* (Hs00208382_m1), *OPA1* (Hs01047013_m1), *ATP5A* (Hs00900735_m1), and *ATP5B* (Hs00969569_m1). All readings were performed in technical duplicate. The relative quantitation was calculated by applying the comparative CT method (2^−ΔΔ^Ct) whereby the mRNA expression levels were normalized against the level of the housekeeping human gene *GAPDH* (ΔCt) with the level of candidate genes in control samples used as the reference (ΔΔCt).

### 4.5. Western Blotting

Cells were washed with PBS and lysed with RIPA lysis buffer (Sigma-Aldrich) containing protease inhibitor cocktail (Sigma-Aldrich). Proteins were denatured in NuPAGE LDS sample buffer and boiled for 5 minutes. 10 *μ*g of proteins was separated through SDS-PAGE using NuPAGE 12% Bis-Tris protein gels in NuPAGE MES SDS running buffer (all from Thermo Fisher Scientific). Proteins were then transferred onto a polyvinylidene difluoride membrane (Amersham Hybond; GE Healthcare Life Sciences, NSW, Australia). The membrane was then blocked with Odyssey blocking buffer (LI-COR Biosciences, NE, USA) for 30 minutes at room temperature. Following successive washes in phosphate-buffered saline containing 0.1% Tween 20 (PBS-T), membranes were incubated with the following primary antibodies diluted in Odyssey blocking buffer: mouse monoclonal ATP5A (1 *μ*g/mL; Abcam), mouse monoclonal ATP5B (2 *μ*g/mL; Abcam), or mouse monoclonal *β*-actin (1 : 1000 dilution; LI-COR Biosciences) at 4°C overnight. After three washes in PBS-T, membranes were incubated with IRDye® 800CW goat anti-mouse (0.05 *μ*g/mL; LI-COR Biosciences) for 60 minutes at room temperature. The membranes were scanned with the Odyssey infrared imaging system (Image Studio, LI-COR Biosciences), and protein band intensity was determined by computerized densitometry (Image Studio Lite, LI-COR Biosciences) and expressed as fold change relative to the control after normalization to *β*-actin.

### 4.6. KINOME*scan*

The KINOME*scan* screening platform (DiscoverX, CA, USA) was employed to measure the interactions of M1 (10 *μ*M in 0.1% DMSO) with 468 kinases covering more than 80% of the human kinome and disease-relevant mutant variants. The KINOME*scan* kinase assay is an ATP-independent active site-directed competition binding assay and thus reports true thermodynamic interaction affinities [47]. The kinase dendrogram was generated using TREEspot software (http://treespot.discoverx.com/). The readout from the KINOME*scan* assay is “percent of control,” where the control is 0.1% DMSO and 100% indicates no inhibition of the kinase by M1 at 10 *μ*M.

### 4.7. mtDNA Copy Number

mtDNA copy numbers were calculated from triplicate reactions of RT-qPCR on 1 ng of total genomic DNA as previously reported [48]. Human iPSCs were harvested using TrypLE Select for DNA isolation using the QIAamp DNA Mini Prep Kit (Qiagen, Chadstone, VIC, Australia) according to the manufacturer's instructions. The mtDNA copy number was determined using the relative copy number method, whereby the average abundance of copies for the mitochondrial genes *ND1*, *ND5*, *ND6*, and *CYB* is given relative to the nuclear gene *GAPDH*/2 to account for the 2 copies of *GAPDH* in the genome. RT-qPCR was carried out using SYBER Green PCR master mix (Sigma-Aldrich). Primers (*GAPDH* F: AGCCACATCGCTCAGACACC, *GAPDH* R: GTACTCAGCGGCAGCATCG; *mtND1* F: GAGCAGTAGCCCAAACAATCTC, *mtND1* R: GGGTCATGATGGCAGGAGTAAT; *mtND5* F: ACATCTGTACCCACGCCTTC, *mtND5* R: CAGGGAGGTAGCGATGAGAG; *mtND6* F: TGGGGTTAGCGATGGAGGTAGG, *mtND6* R: AATAGGATCCTCCCGAATCAAC; and *mtCYB* F: CTGATCCTCCAAATCACCACAG, *mtCYB* R: GCGCCATTGGCGTGAAGGTA) were purchased from GeneWorks (Thebarton, SA, Australia).

### 4.8. Statistics

All values are expressed as mean ± standard error of the mean (SEM). Significance of the differences was evaluated using un/paired Student's *t*-test or one-way paired ANOVA followed by Dunnett's multiple comparison post hoc analysis where appropriate. *P* < 0.05 is considered statistically significant.

## Data Availability

All data generated or analyzed during this study are included in this published article (and its supplementary information files).

## Supplementary Materials

Supplementary Materials Supplementary Figure 1: gene expression of endoderm and ectoderm markers in human iPSCs (iPSC-Foreskin-2 cell line) treated with M1 for 48 hours. Supplementary Figure 2: effect of M1 in CERA007c6 iPSCs. Supplementary Figure 3: gene expression of human iPSCs (iPSC-Foreskin-2 cell line) cultured in differentiation medium for 48 hours. Supplementary Figure 4: gene expression of ATP synthase subunits in human iPSCs cultured in 2D and 3D formats. Supplementary Table 1: kinase profiling of M1 at 10 *μ*M by the KINOME*scan* assay.

## References

[1] Verma V., Purnamawati K., Manasi, Shim W. (2013). Steering signal transduction pathway towards cardiac lineage from human pluripotent stem cells: a review. *Cellular Signalling*.

[2] Chen H., Chan D. C. (2017). Mitochondrial dynamics in regulating the unique phenotypes of cancer and stem cells. *Cell Metabolism*.

[3] Lees J. G., Gardner D. K., Harvey A. J. (2017). Pluripotent stem cell metabolism and mitochondria: beyond ATP. *Stem Cells International*.

[4] Seo B., Yoon S., Do J. (2018). Mitochondrial dynamics in stem cells and differentiation. *International Journal of Molecular Sciences*.

[5] Wanet A., Arnould T., Najimi M., Renard P. (2015). Connecting mitochondria, metabolism, and stem cell fate. *Stem Cells and Development*.

[6] Varum S., Rodrigues A. S., Moura M. B. (2011). Energy metabolism in human pluripotent stem cells and their differentiated counterparts. *PLoS One*.

[7] Hoque A., Sivakumaran P., Bond S. T. (2018). Mitochondrial fission protein Drp1 inhibition promotes cardiac mesodermal differentiation of human pluripotent stem cells. *Cell Death Discovery*.

[8] Cliff T. S., Wu T., Boward B. R. (2017). MYC controls human pluripotent stem cell fate decisions through regulation of metabolic flux. *Cell Stem Cell*.

[9] Zhang J., Khvorostov I., Hong J. S. (2011). UCP2 regulates energy metabolism and differentiation potential of human pluripotent stem cells. *The EMBO Journal*.

[10] Spyrou J., Gardner D. K., Harvey A. J. (2019). Metabolomic and transcriptional analyses reveal atmospheric oxygen during human induced pluripotent stem cell generation impairs metabolic reprogramming. *Stem Cells*.

[11] Lees J. G., Cliff T. S., Gammilonghi A. (2019). Oxygen regulates human pluripotent stem cell metabolic flux. *Stem Cells International*.

[12] Richard A., Vallin E., Romestaing C., Roussel D., Gandrillon O., Gonin-Giraud S. (2019). Erythroid differentiation displays a peak of energy consumption concomitant with glycolytic metabolism rearrangements. *PLoS One*.

[13] Lees J. G., Gardner D. K., Harvey A. J. (2018). Mitochondrial and glycolytic remodeling during nascent neural differentiation of human pluripotent stem cells. *Development*.

[14] St John J. C., Ramalho-Santos J., Gray H. L. (2005). The expression of mitochondrial DNA transcription factors during early cardiomyocyte in vitro differentiation from human embryonic stem cells. *Cloning and Stem Cells*.

[15] Chan D. C. (2012). Fusion and fission: interlinked processes critical for mitochondrial health. *Annual Review of Genetics*.

[16] Knowlton A. A., Chen L., Malik Z. A. (2014). Heart failure and mitochondrial dysfunction: the role of mitochondrial fission/fusion abnormalities and new therapeutic strategies. *Journal of Cardiovascular Pharmacology*.

[17] Dorn G. W. (2015). Mitochondrial dynamism and heart disease: changing shape and shaping change. *EMBO Molecular Medicine*.

[18] Chen Y., Dorn G. W. (2013). PINK1-phosphorylated mitofusin 2 is a parkin receptor for culling damaged mitochondria. *Science*.

[19] Kasahara A., Cipolat S., Chen Y., Dorn G. W., Scorrano L. (2013). Mitochondrial fusion directs cardiomyocyte differentiation via calcineurin and Notch signaling. *Science*.

[20] Wang D., Wang J., Bonamy G. M. C. (2012). A small molecule promotes mitochondrial fusion in mammalian cells. *Angewandte Chemie International Edition*.

[21] Asalla S., Girada S. B., Kuna R. S. (2016). Restoring mitochondrial function: a small molecule-mediated approach to enhance glucose stimulated insulin secretion in cholesterol accumulated pancreatic beta cells. *Scientific Reports*.

[22] Asalla S., Mohareer K., Banerjee S. (2017). Small molecule mediated restoration of mitochondrial function augments anti-mycobacterial activity of human macrophages subjected to cholesterol induced asymptomatic dyslipidemia. *Frontiers in Cellular and Infection Microbiology*.

[23] Hung C. H.-L., Cheng S. S. Y., Cheung Y. T. (2018). A reciprocal relationship between reactive oxygen species and mitochondrial dynamics in neurodegeneration. *Redox Biology*.

[24] Mandal S., Lindgren A. G., Srivastava A. S., Clark A. T., Banerjee U. (2011). Mitochondrial function controls proliferation and early differentiation potential of embryonic stem cells. *Stem Cells*.

[25] Folmes C. D. L., Nelson T. J., Martinez-Fernandez A. (2011). Somatic oxidative bioenergetics transitions into pluripotency-dependent glycolysis to facilitate nuclear reprogramming. *Cell Metabolism*.

[26] Todd L. R., Damin M. N., Gomathinayagam R., Horn S. R., Means A. R., Sankar U. (2010). Growth factor *erv1*-like modulates Drp1 to preserve mitochondrial dynamics and function in mouse embryonic stem cells. *Molecular Biology of the Cell*.

[27] Cho Y. M., Kwon S., Pak Y. K. (2006). Dynamic changes in mitochondrial biogenesis and antioxidant enzymes during the spontaneous differentiation of human embryonic stem cells. *Biochemical and Biophysical Research Communications*.

[28] Zhou W., Choi M., Margineantu D. (2012). HIF1*α* induced switch from bivalent to exclusively glycolytic metabolism during ESC-to-EpiSC/hESC transition. *The EMBO Journal*.

[29] Spyrou J., Gardner D. K., Harvey A. J. (2019). Metabolism is a key regulator of induced pluripotent stem cell reprogramming. *Stem Cells International*.

[30] Cliff T. S., Dalton S. (2017). Metabolic switching and cell fate decisions: implications for pluripotency, reprogramming and development. *Current Opinion in Genetics & Development*.

[31] Facucho-Oliveira J. M., Alderson J., Spikings E. C., Egginton S., St John J. C. (2007). Mitochondrial DNA replication during differentiation of murine embryonic stem cells. *Journal of Cell Science*.

[32] Chung S., Arrell D. K., Faustino R. S., Terzic A., Dzeja P. P. (2010). Glycolytic network restructuring integral to the energetics of embryonic stem cell cardiac differentiation. *Journal of Molecular and Cellular Cardiology*.

[33] Liau B., Christoforou N., Leong K. W., Bursac N. (2011). Pluripotent stem cell-derived cardiac tissue patch with advanced structure and function. *Biomaterials*.

[34] Zhang M., Schulte J. S., Heinick A. (2015). Universal cardiac induction of human pluripotent stem cells in two and three-dimensional formats: implications for in vitro maturation. *Stem Cells*.

[35] Chung S., Dzeja P. P., Faustino R. S., Perez-Terzic C., Behfar A., Terzic A. (2007). Mitochondrial oxidative metabolism is required for the cardiac differentiation of stem cells. *Nature Clinical Practice Cardiovascular Medicine*.

[36] Trotta A. P., Chipuk J. E. (2017). Mitochondrial dynamics as regulators of cancer biology. *Cellular and Molecular Life Sciences*.

[37] Seo H., Lee I., Chung H. S. (2016). ATP5B regulates mitochondrial fission and fusion in mammalian cells. *Animal Cells and Systems*.

[38] Givvimani S., Pushpakumar S. B., Metreveli N., Veeranki S., Kundu S., Tyagi S. C. (2015). Role of mitochondrial fission and fusion in cardiomyocyte contractility. *International Journal of Cardiology*.

[39] Liesa M., Borda-d'Água B., Medina-Gómez G. (2008). Mitochondrial fusion is increased by the nuclear coactivator PGC-1*β*. *PLoS One*.

[40] Khacho M., Clark A., Svoboda D. S. (2016). Mitochondrial dynamics impacts stem cell identity and fate decisions by regulating a nuclear transcriptional program. *Cell Stem Cell*.

[41] Guan X., Wang L., Liu Z. (2016). miR-106a promotes cardiac hypertrophy by targeting mitofusin 2. *Journal of Molecular and Cellular Cardiology*.

[42] Sun Y. L., Li S. H., Yang L., Wang Y. (2018). miR-376b-3p attenuates mitochondrial fission and cardiac hypertrophy by targeting mitochondrial fission factor. *Clinical and Experimental Pharmacology and Physiology*.

[43] Yu J., Vodyanik M. A., Smuga-Otto K. (2007). Induced pluripotent stem cell lines derived from human somatic cells. *Science*.

[44] Lim S. Y., Sivakumaran P., Crombie D. E., Dusting G. J., Pébay A., Dilley R. J. (2013). Trichostatin A enhances differentiation of human induced pluripotent stem cells to cardiogenic cells for cardiac tissue engineering. *Stem Cells Translational Medicine*.

[45] Hernández D., Millard R., Sivakumaran P. (2016). Electrical stimulation promotes cardiac differentiation of human induced pluripotent stem cells. *Stem Cells International*.

[46] Crombie D. E., Curl C. L., Raaijmakers A. J. A. (2017). Friedreich’s ataxia induced pluripotent stem cell-derived cardiomyocytes display electrophysiological abnormalities and calcium handling deficiency. *Aging*.

[47] Davis M. I., Hunt J. P., Herrgard S. (2011). Comprehensive analysis of kinase inhibitor selectivity. *Nature Biotechnology*.

[48] Lees J. G., Rathjen J., Sheedy J. R., Gardner D. K., Harvey A. J. (2015). Distinct profiles of human embryonic stem cell metabolism and mitochondria identified by oxygen. *Reproduction*.

