# Supplementary material for: Mitochondrial Fusion by M1 Promotes Embryoid Body Cardiac Differentiation of Human Pluripotent Stem Cells
Source: Stem Cells Int. 2019 Sep 19;2019:6380135. doi: 10.1155/2019/6380135 (PMC6770295; doi:10.1155/2019/6380135)
Supplement: Supplementary Materials — Supplementary Figure 1: gene expression of endoderm and ectoderm markers in human iPSCs (iPSC-Foreskin-2 cell line) treated with M1 for 48 hours. Supplementary Figure 2: effect of M1 in CERA007c6 iPSCs. Supplementary Figure 3: gene expression of human iPSCs (iPSC-Foreskin-2 cell line) cultured in differentiation medium for 48 hours. Supplementary Figure 4: gene expression of ATP synthase subunits in human iPSCs cultured in 2D and 3D formats. Supplementary Table 1: kinase profiling of M1 at 10 μM by the KINOMEscan assay. [file 6380135.f1.docx]

# Supplementary materials


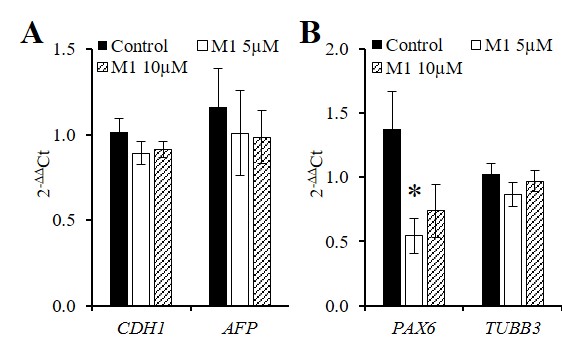


**Supplementary Figure 1. (A-B)** mRNA expression of endoderm **(A)** and ectoderm markers **(B)** in human iPSCs (iPSC-Foreskin-2 cell line) treated with either DMSO (control) or M1 at 5 or 10 µM for 48 hours (n = 7). Data are expressed as mean ± SEM. **P* < 0.05 vs. control by one-way paired ANOVA with the Dunnett’s post hoc test.


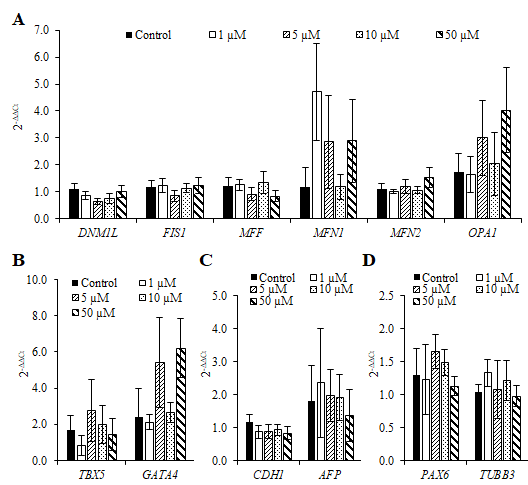


**Supplementary Figure 2. Effect of M1 in CERA007c6 iPSCs. (A-D)** mRNA expression of mitochondrial fission and fusion markers **(A)**, mesodermal cardiac transcription factors **(B),** endoderm marker **(C)** and ectoderm marker **(D)** in human iPSCs treated with either DMSO (control) or M1 at 1, 5, 10 or 50 µM for 48 hours (n = 6). Data are expressed as mean ± SEM. **P* < 0.05 vs. control by one-way paired ANOVA with the Dunnett’s post hoc test.

**
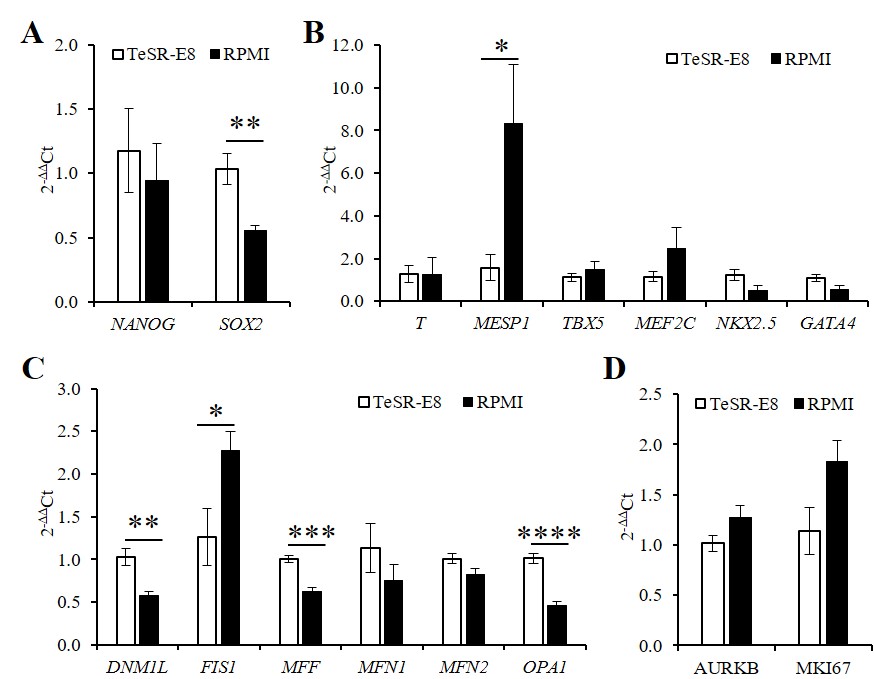
**

**Supplementary Figure 3. Gene expression of human iPSCs (iPSC-Foreskin-2 cell line) cultured in differentiation medium for 48 hours. (A-D)** mRNA expression of pluripotency markers **(A)**, mesodermal cardiac transcription factors **(B)**, mitochondrial fission and fusion markers **(C)** and cell proliferation markers **(D)** in human iPSCs cultured in TeSR-E8 and RPMI+B-27 medium for 48 hours. n = 5. Data are expressed as mean ± SEM. *P < 0.05, **P < 0.01, ***P < 0.001, ****P < 0.0001 vs. control by an unpaired Student’s t-test.

**
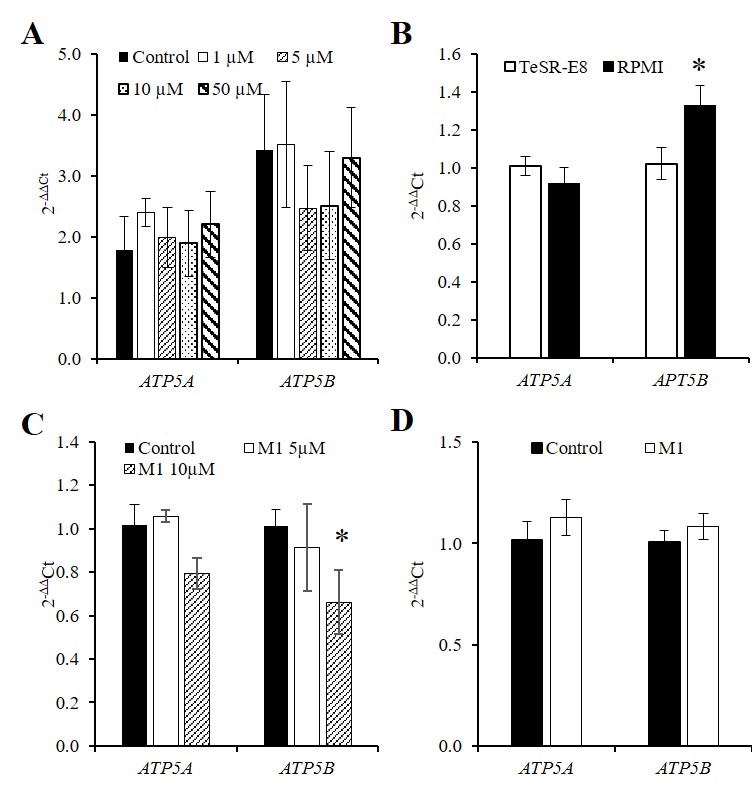
**

**Supplementary Figure 4. Gene expression of ATP synthase subunits in human iPSCs cultured in 2D and 3D formats.** **(A)** mRNA levels of ATP synthase subunits in CERA007c6 iPSCs cultured in TeSR-E8 and treated with either DMSO (control) or M1 for 48 hours (n = 6). **(B-C)** mRNA levels of ATP synthase subunits in iPSCs (iPSC-Foreskin-2 cell line) cultured in TeSR-E8 or RPMI+B-27 (RPMI) medium (n = 5-7) **(B)**, and following M1 treatment in RPMI+B-27 medium for 48 hours (n = 7) **(C)**. **(D)** mRNA levels of ATP synthase subunits in iPSCs (iPSC-Foreskin-2 cell line) treated with DMSO (control) or 5 µM M1 for 6 days during EB formation (n = 4). Data are expressed as mean ± SEM. *P < 0.05 vs. control by unpaired Student’s t-test **(B)**, and one-way ANOVA with the Dunnett’s post hoc test **(C)**.

**Supplementary Table 1.** Kinase profiling of M1 at 10 uM by KINOMEscan assay.

| DiscoveRx Gene Symbol | Percent Control | DiscoveRx Gene Symbol | Percent Control |
| --- | --- | --- | --- |
| AAK1 | 92 | BRAF | 100 |
| ABL1(E255K)-phosphorylated | 47 | BRAF(V600E) | 79 |
| ABL1(F317I)-nonphosphorylated | 99 | BRK | 85 |
| ABL1(F317I)-phosphorylated | 90 | BRSK1 | 94 |
| ABL1(F317L)-nonphosphorylated | 92 | BRSK2 | 95 |
| ABL1(F317L)-phosphorylated | 79 | BTK | 84 |
| ABL1(H396P)-nonphosphorylated | 92 | BUB1 | 100 |
| ABL1(H396P)-phosphorylated | 52 | CAMK1 | 84 |
| ABL1(M351T)-phosphorylated | 85 | CAMK1B | 66 |
| ABL1(Q252H)-nonphosphorylated | 67 | CAMK1D | 92 |
| ABL1(Q252H)-phosphorylated | 61 | CAMK1G | 89 |
| ABL1(T315I)-nonphosphorylated | 89 | CAMK2A | 96 |
| ABL1(T315I)-phosphorylated | 76 | CAMK2B | 100 |
| ABL1(Y253F)-phosphorylated | 57 | CAMK2D | 91 |
| ABL1-nonphosphorylated | 74 | CAMK2G | 91 |
| ABL1-phosphorylated | 53 | CAMK4 | 93 |
| ABL2 | 97 | CAMKK1 | 87 |
| ACVR1 | 97 | CAMKK2 | 82 |
| ACVR1B | 76 | CASK | 92 |
| ACVR2A | 100 | CDC2L1 | 100 |
| ACVR2B | 100 | CDC2L2 | 100 |
| ACVRL1 | 97 | CDC2L5 | 86 |
| ADCK3 | 97 | CDK11 | 78 |
| ADCK4 | 97 | CDK2 | 89 |
| AKT1 | 91 | CDK3 | 97 |
| AKT2 | 92 | CDK4 | 85 |
| AKT3 | 100 | CDK4-cyclinD1 | 100 |
| ALK | 90 | CDK4-cyclinD3 | 68 |
| ALK(C1156Y) | 86 | CDK5 | 99 |
| ALK(L1196M) | 86 | CDK7 | 89 |
| AMPK-alpha1 | 83 | CDK8 | 100 |
| AMPK-alpha2 | 83 | CDK9 | 100 |
| ANKK1 | 92 | CDKL1 | 74 |
| ARK5 | 100 | CDKL2 | 97 |
| ASK1 | 93 | CDKL3 | 82 |
| ASK2 | 84 | CDKL5 | 81 |
| AURKA | 88 | CHEK1 | 97 |
| AURKB | 93 | CHEK2 | 89 |
| AURKC | 99 | CIT | 92 |
| AXL | 97 | CLK1 | 95 |
| BIKE | 94 | CLK2 | 100 |
| BLK | 87 | CLK3 | 93 |
| BMPR1A | 92 | CLK4 | 83 |
| BMPR1B | 87 | CSF1R | 92 |
| BMPR2 | 84 | CSF1R-autoinhibited | 98 |
| BMX | 99 | CSK | 96 |
| CSNK1A1 | 81 | EPHB4 | 91 |
| CSNK1A1L | 99 | EPHB6 | 88 |
| CSNK1D | 89 | ERBB2 | 100 |
| CSNK1E | 91 | ERBB3 | 91 |
| CSNK1G1 | 97 | ERBB4 | 85 |
| CSNK1G2 | 92 | ERK1 | 96 |
| CSNK1G3 | 84 | ERK2 | 100 |
| CSNK2A1 | 91 | ERK3 | 85 |
| CSNK2A2 | 79 | ERK4 | 100 |
| CTK | 98 | ERK5 | 100 |
| DAPK1 | 97 | ERK8 | 97 |
| DAPK2 | 97 | ERN1 | 84 |
| DAPK3 | 90 | FAK | 95 |
| DCAMKL1 | 68 | FER | 92 |
| DCAMKL2 | 100 | FES | 96 |
| DCAMKL3 | 95 | FGFR1 | 83 |
| DDR1 | 98 | FGFR2 | 100 |
| DDR2 | 92 | FGFR3 | 100 |
| DLK | 99 | FGFR3(G697C) | 100 |
| DMPK | 100 | FGFR4 | 87 |
| DMPK2 | 92 | FGR | 83 |
| DRAK1 | 100 | FLT1 | 81 |
| DRAK2 | 100 | FLT3 | 99 |
| DYRK1A | 82 | FLT3(D835H) | 96 |
| DYRK1B | 71 | FLT3(D835V) | 51 |
| DYRK2 | 82 | FLT3(D835Y) | 82 |
| EGFR | 100 | FLT3(ITD) | 73 |
| EGFR(E746-A750del) | 91 | FLT3(ITD,D835V) | 93 |
| EGFR(G719C) | 71 | FLT3(ITD,F691L) | 66 |
| EGFR(G719S) | 60 | FLT3(K663Q) | 97 |
| EGFR(L747-E749del, A750P) | 99 | FLT3(N841I) | 100 |
| EGFR(L747-S752del, P753S) | 93 | FLT3(R834Q) | 97 |
| EGFR(L747-T751del,Sins) | 68 | FLT3-autoinhibited | 96 |
| EGFR(L858R) | 100 | FLT4 | 100 |
| EGFR(L858R,T790M) | 82 | FRK | 86 |
| EGFR(L861Q) | 67 | FYN | 93 |
| EGFR(S752-I759del) | 55 | GAK | 95 |
| EGFR(T790M) | 83 | GCN2(Kin.Dom.2,S808G) | 90 |
| EIF2AK1 | 78 | GRK1 | 98 |
| EPHA1 | 93 | GRK2 | 100 |
| EPHA2 | 100 | GRK3 | 95 |
| EPHA3 | 99 | GRK4 | 87 |
| EPHA4 | 97 | GRK7 | 100 |
| EPHA5 | 98 | GSK3A | 94 |
| EPHA6 | 97 | GSK3B | 82 |
| EPHA7 | 99 | HASPIN | 93 |
| EPHA8 | 100 | HCK | 92 |
| EPHB1 | 95 | HIPK1 | 90 |
| EPHB2 | 100 | HIPK2 | 97 |
| EPHB3 | 99 | HIPK3 | 87 |
| HIPK4 | 88 | MAP4K4 | 100 |
| HPK1 | 100 | MAP4K5 | 100 |
| HUNK | 100 | MAPKAPK2 | 79 |
| ICK | 100 | MAPKAPK5 | 96 |
| IGF1R | 99 | MARK1 | 99 |
| IKK-alpha | 95 | MARK2 | 87 |
| IKK-beta | 100 | MARK3 | 97 |
| IKK-epsilon | 98 | MARK4 | 100 |
| INSR | 100 | MAST1 | 78 |
| INSRR | 100 | MEK1 | 92 |
| IRAK1 | 95 | MEK2 | 94 |
| IRAK3 | 94 | MEK3 | 74 |
| IRAK4 | 80 | MEK4 | 98 |
| ITK | 95 | MEK5 | 87 |
| JAK1(JH1domain-catalytic) | 100 | MEK6 | 94 |
| JAK1(JH2domain-pseudokinase) | 100 | MELK | 85 |
| JAK2(JH1domain-catalytic) | 70 | MERTK | 84 |
| JAK3(JH1domain-catalytic) | 91 | MET | 100 |
| JNK1 | 100 | MET(M1250T) | 81 |
| JNK2 | 94 | MET(Y1235D) | 79 |
| JNK3 | 90 | MINK | 86 |
| KIT | 97 | MKK7 | 94 |
| KIT(A829P) | 90 | MKNK1 | 88 |
| KIT(D816H) | 94 | MKNK2 | 77 |
| KIT(D816V) | 96 | MLCK | 100 |
| KIT(L576P) | 99 | MLK1 | 100 |
| KIT(V559D) | 98 | MLK2 | 95 |
| KIT(V559D,T670I) | 93 | MLK3 | 92 |
| KIT(V559D,V654A) | 100 | MRCKA | 97 |
| KIT-autoinhibited | 77 | MRCKB | 94 |
| LATS1 | 95 | MST1 | 84 |
| LATS2 | 99 | MST1R | 97 |
| LCK | 90 | MST2 | 99 |
| LIMK1 | 92 | MST3 | 97 |
| LIMK2 | 96 | MST4 | 96 |
| LKB1 | 100 | MTOR | 98 |
| LOK | 77 | MUSK | 98 |
| LRRK2 | 96 | MYLK | 82 |
| LRRK2(G2019S) | 85 | MYLK2 | 73 |
| LTK | 85 | MYLK4 | 96 |
| LYN | 100 | MYO3A | 97 |
| LZK | 93 | MYO3B | 100 |
| MAK | 84 | NDR1 | 80 |
| MAP3K1 | 87 | NDR2 | 100 |
| MAP3K15 | 77 | NEK1 | 100 |
| MAP3K2 | 93 | NEK10 | 96 |
| MAP3K3 | 99 | NEK11 | 100 |
| MAP3K4 | 89 | NEK2 | 97 |
| MAP4K2 | 86 | NEK3 | 94 |
| MAP4K3 | 98 | NEK4 | 89 |
| NEK5 | 93 | PIP5K1A | 89 |
| NEK6 | 100 | PIP5K1C | 83 |
| NEK7 | 98 | PIP5K2B | 100 |
| NEK9 | 100 | PIP5K2C | 76 |
| NIK | 92 | PKAC-alpha | 95 |
| NIM1 | 90 | PKAC-beta | 84 |
| NLK | 85 | PKMYT1 | 85 |
| OSR1 | 87 | PKN1 | 100 |
| p38-alpha | 97 | PKN2 | 97 |
| p38-beta | 83 | PKNB(M.tuberculosis) | 79 |
| p38-delta | 88 | PLK1 | 90 |
| p38-gamma | 91 | PLK2 | 92 |
| PAK1 | 84 | PLK3 | 89 |
| PAK2 | 83 | PLK4 | 84 |
| PAK3 | 93 | PRKCD | 89 |
| PAK4 | 88 | PRKCE | 83 |
| PAK6 | 98 | PRKCH | 80 |
| PAK7 | 97 | PRKCI | 62 |
| PCTK1 | 90 | PRKCQ | 87 |
| PCTK2 | 100 | PRKD1 | 100 |
| PCTK3 | 100 | PRKD2 | 100 |
| PDGFRA | 97 | PRKD3 | 100 |
| PDGFRB | 92 | PRKG1 | 89 |
| PDPK1 | 98 | PRKG2 | 61 |
| PFCDPK1(P.falciparum) | 81 | PRKR | 94 |
| PFPK5(P.falciparum) | 98 | PRKX | 89 |
| PFTAIRE2 | 100 | PRP4 | 96 |
| PFTK1 | 100 | PYK2 | 95 |
| PHKG1 | 91 | QSK | 78 |
| PHKG2 | 97 | RAF1 | 100 |
| PIK3C2B | 79 | RET | 96 |
| PIK3C2G | 88 | RET(M918T) | 70 |
| PIK3CA | 91 | RET(V804L) | 67 |
| PIK3CA(C420R) | 80 | RET(V804M) | 97 |
| PIK3CA(E542K) | 82 | RIOK1 | 89 |
| PIK3CA(E545A) | 94 | RIOK2 | 93 |
| PIK3CA(E545K) | 75 | RIOK3 | 100 |
| PIK3CA(H1047L) | 97 | RIPK1 | 99 |
| PIK3CA(H1047Y) | 94 | RIPK2 | 98 |
| PIK3CA(I800L) | 100 | RIPK4 | 100 |
| PIK3CA(M1043I) | 100 | RIPK5 | 80 |
| PIK3CA(Q546K) | 83 | ROCK1 | 77 |
| PIK3CB | 93 | ROCK2 | 83 |
| PIK3CD | 92 | ROS1 | 93 |
| PIK3CG | 94 | RPS6KA4(Kin.Dom.1-N-terminal) | 62 |
| PIK4CB | 97 | RPS6KA4(Kin.Dom.2-C-terminal) | 95 |
| PIKFYVE | 92 | RPS6KA5(Kin.Dom.1-N-terminal) | 84 |
| PIM1 | 78 | RPS6KA5(Kin.Dom.2-C-terminal) | 98 |
| PIM2 | 95 | RSK1(Kin.Dom.1-N-terminal) | 100 |
| PIM3 | 78 | RSK1(Kin.Dom.2-C-terminal) | 93 |
| RSK2(Kin.Dom.1-N-terminal) | 90 | TIE2 | 73 |
| RSK2(Kin.Dom.2-C-terminal) | 95 | TLK1 | 95 |
| RSK3(Kin.Dom.1-N-terminal) | 98 | TLK2 | 94 |
| RSK3(Kin.Dom.2-C-terminal) | 97 | TNIK | 100 |
| RSK4(Kin.Dom.1-N-terminal) | 82 | TNK1 | 71 |
| RSK4(Kin.Dom.2-C-terminal) | 87 | TNK2 | 86 |
| S6K1 | 82 | TNNI3K | 94 |
| SBK1 | 91 | TRKA | 71 |
| SGK | 79 | TRKB | 99 |
| SgK110 | 85 | TRKC | 87 |
| SGK2 | 87 | TRPM6 | 78 |
| SGK3 | 92 | TSSK1B | 81 |
| SIK | 98 | TSSK3 | 93 |
| SIK2 | 93 | TTK | 96 |
| SLK | 89 | TXK | 93 |
| SNARK | 74 | TYK2(JH1domain-catalytic) | 67 |
| SNRK | 78 | TYK2(JH2domain-pseudokinase) | 92 |
| SRC | 96 | TYRO3 | 93 |
| SRMS | 70 | ULK1 | 89 |
| SRPK1 | 100 | ULK2 | 94 |
| SRPK2 | 100 | ULK3 | 92 |
| SRPK3 | 90 | VEGFR2 | 86 |
| STK16 | 97 | VPS34 | 88 |
| STK33 | 95 | VRK2 | 90 |
| STK35 | 96 | WEE1 | 100 |
| STK36 | 100 | WEE2 | 93 |
| STK39 | 66 | WNK1 | 100 |
| SYK | 94 | WNK2 | 97 |
| TAK1 | 86 | WNK3 | 100 |
| TAOK1 | 87 | WNK4 | 100 |
| TAOK2 | 78 | YANK1 | 75 |
| TAOK3 | 85 | YANK2 | 76 |
| TBK1 | 96 | YANK3 | 90 |
| TEC | 99 | YES | 90 |
| TESK1 | 88 | YSK1 | 100 |
| TGFBR1 | 100 | YSK4 | 84 |
| TGFBR2 | 100 | ZAK | 92 |
| TIE1 | 100 | ZAP70 | 82 |
